# Intravascular lithotripsy (IVL) enabled the percutaneous closure of a severely calcified paravalvular leak regurgitation following implantation of a self-expandable transcatheter aortic valve: a case report

**DOI:** 10.3389/fcvm.2024.1359711

**Published:** 2024-02-21

**Authors:** Salvatore Evola, Alessandro D’Agostino, Daniele Adorno, Oreste Fabio Triolo, Gioacchino Giarratana, Sebastiano Castrovinci, Vincenzo Argano, Eustaquio Maria Onorato

**Affiliations:** ^1^ Catheterization Laboratory, Department of Medicine and Cardiology, Azienda Ospedaliera Universitaria Policlinico “P. Giaccone”, Palermo, Italy; ^2^ U.O.C. Cardiochirurgia, Azienda Ospedaliera Universitaria Policlinico “P. Giaccone”, Palermo, Italy; ^3^ University Cardiology Department, IRCCS Galeazzi-Sant’Ambrogio Hospital, Milan, Italy

**Keywords:** paravalvular leak regurgitation, calcified paravalvular leak, self-expandable transcatheter aortic valve implantation, paravalvular leak closure, intravascular lithotripsy

## Abstract

**Background:**

Closure of paravalvular leak (PVL) regurgitation after self-expandable (SE) transcatheter aortic valve implantation (TAVI) may be more challenging than after balloon-expandable (BE) valve implantation.

**Case summary:**

An 85-year-old woman suffering from long-standing atrial fibrillation and severe symptomatic aortic stenosis underwent SE TAVI (26 mm Evolut™ R®, Medtronic Inc., MN, USA). A total of eighteen months after TAVI she was admitted for congestive heart failure and two-dimensional (2D) transesophageal echocardiography (TEE) color Doppler showed moderate-severe PVL regurgitation due to a long and heavily calcified leak located below the left coronary sinus. The patient was deemed to be at prohibitive surgical risk and a catheter-based PVL closure procedure was planned. A first attempt to cross the PVL from the femoral artery was unsuccessful due to an inappropriate angle between the catheter and the entry site of this hard-to-approach calcified leak. A Terumo hydrophilic guidewire 0.35 inch-260 cm from the right radial artery was then successfully advanced across the leak to the left ventricle (LV); however, of most of the catheters used, only a Glidecath 4-Fr could cross the leak over the hydrophilic wire. The hydrophilic guidewire was replaced with a stiffer guidewire that, after creating a loop in the LV, was advanced across the self-expandable valve into the descending aorta where it was snared and externalized through the left femoral artery, thus creating an arterio-arterial (AA) loop. A 6-Fr Multipurpose guiding catheter was advanced over the exchange wire and the leak was crossed with an additional 0.0014 coronary guidewire (PILOT, Abbott Vascular), predilated with two non-compliant balloon dilatation catheters, and finally, the PVL was engaged with a 3.0 mm × 12 mm Shockwave balloon (Shockwave Medical Inc, Santa Clara, California, USA). Intravascular lithotripsy (IVL) application to this highly calcified leak and the increased support provided by the stiff guidewire finally allowed the progression of the 6-Fr dedicated delivery sheath (ODS III) into the LV. A 5 mm square twist (ST) device (PLD, Occlutech, Helsingborg, Sweden) was successfully deployed within the leak and the final echocardiographic and angiographic control confirmed the effective PVL closure.

**Discussion:**

In patients at high surgical risk with moderate to severe regurgitation after SE TAVI due to a hard-to-approach calcified long tract, an extra AA support loop is mandatory during percutaneous PVL closure. Furthermore, IVL application greatly facilitates the progression of the delivery sheath and occluder which is key to a successful procedure.

## Highlights

• Paravalvular leak (PVL) with moderate to severe regurgitation after transcatheter aortic valve implantation (TAVI) is a dreaded complication associated with high rates of morbidity and mortality.
• PVL closure after self-expandable (SE) TAVI is more technically challenging than after balloon-expandable (BE) valve implantation.
• The most challenging problems are represented by multiple or irregular leaks, low crossing positions over frame struts, high sealing skirts on SE valves, and calcified long leaks.
• This report is the first successful lithotripsy implementation for the treatment of a severely calcified long leakage with severe regurgitation after 26 mm SE Evolut™ R®).

## Introduction

PVL closure after SE TAVI may be more challenging than after balloon-expandable valve implantation, as strut avoidance is much more difficult (1, 2). A high burden of calcium may persist in the TAVI device landing zone, limiting equipment accessibility and navigation between the prosthesis frame and native tissue (3). For anatomically hard-to-approach long calcified tracts, the use of a radial approach and the creation of an extra AA support loop can greatly facilitate the closure procedure (4–6).

## Case presentation

An 85-year-old woman, suffering from hypertension and dyslipidemia was admitted for congestive heart failure (NYHA class III). The patient's medical history was notable for long-standing atrial fibrillation and severe symptomatic aortic stenosis that was treated with a 26 mm SE Evolut™ R® in April 2022 at another center. Nonobstructive coronary artery disease was documented and the patient was found to be on optimal medical therapy (omeprazole 20 mg, allopurinol 300 mg, edoxaban 30 mg, furosemide 25 mg, bisoprolol 1.25 mg). A few days after TAVI, a cardiac resynchronization therapy device (CRT) implantation was implanted, which was complicated by infection, decubitus ulcer, and left upper limb thrombosis, requiring explantation of the CRT.

**Figure 1:**
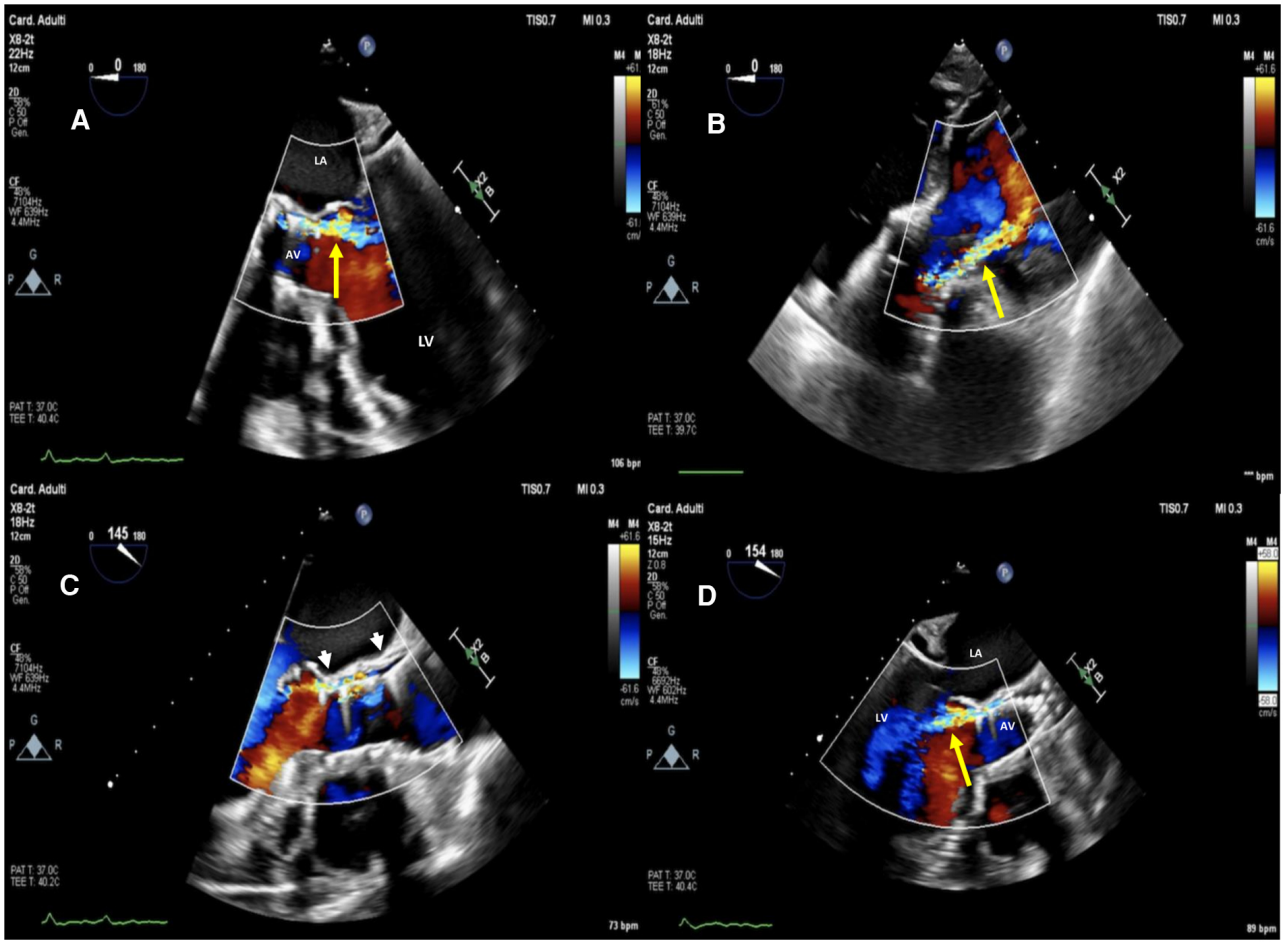
Baseline two-dimensional (2D) transesophageal echocardiography (TEE) color Doppler at mid-esophageal 5 chamber view (**A**), at deep transgastric 5 chamber view (**B**) and mid-esophageal AV long-axis view (**C,D**) showing a 26 mm SE evolut™ R® with moderate to severe regurgitation (yellow arrow) through a long, tortuous paravalvular leakage below the left coronary sinus (white arrowheads). La, left atrium; LV, left ventricle; AV, aortic valve.

**Figure 2:**
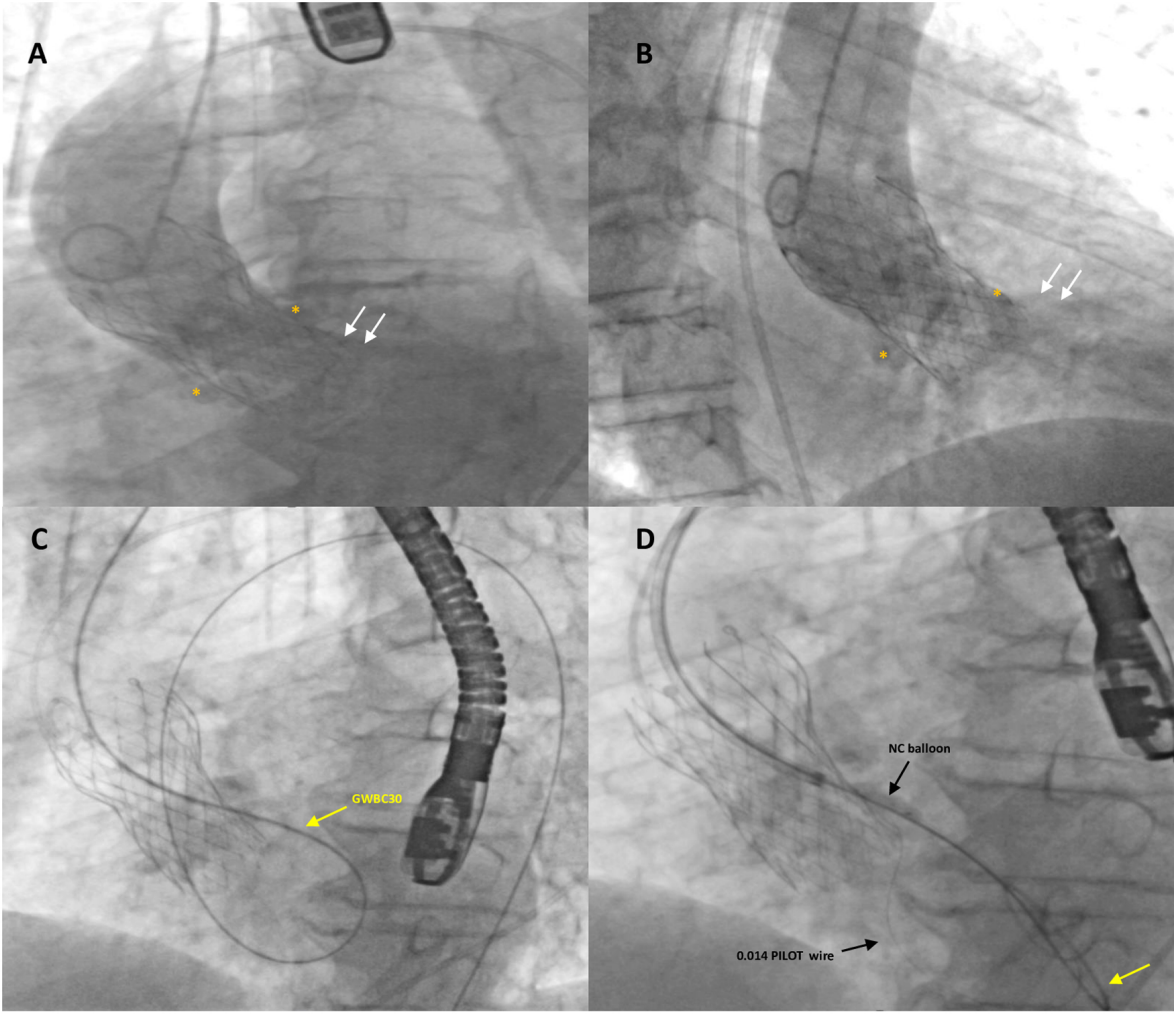
Fluoro-angiographic intra-procedural procedural steps (1). Left anterior oblique (**A**) and right anterior oblique (**B**) aortography with a Pig-tail 5-Fr catheter showing severe regurgitation (white arrows) through a highly calcified paravalvular leak of a self-expandable transcatheter aortic valve (TAVI) (26 mm Evolut™ R®); white asterisks indicate the large calcific nodules in the annular region. The stiff guidewire (GWBC30, Medtronic Confida™) (yellow arrow) from the aorta crossed the tortuous calcified leak retrogradely and, after forming a loop in the left ventricle, was advanced over the TAVI prosthetic leaflets into the descending aorta where it was snared and externalized through the left femoral artery, thus creating an arterio-arterial loop (**C**). A 6-Fr Multipurpose guiding catheter was advanced over the exchange stiff wire to the entry of the leak. Subsequently, the leak was crossed with an additional 0.0014 coronary guidewire (PILOT, Abbott Vascular) advanced into the left ventricle and then into the aorta and predilated with non-compliant (NC) balloon dilatation catheters (**D**).

2D transesophageal echocardiography (TEE) color Doppler showed moderate residual PVL regurgitation shortly after TAVI implantation. Over time, the residual regurgitation increased to moderate to severe through a 10 mm long and 4 mm in diameter, tortuous, heavily calcified leak located below the left coronary sinus (Figure 1, Supplementary Videos S1–S3). The patient declined to redo surgery and after discussion with the heart team, transcatheter PVL closure was planned. An informed consent form was signed by the patient. The procedure was performed under general anesthesia and TEE/angio-fluoroscopic guidance. A 6-Fr multipurpose (MP) catheter was advanced from the right femoral artery and a 5-Fr pigtail angiographic catheter from the right radial artery into the ascending aorta. A first attempt to cross the PVL was unsuccessful due to an inappropriate angle between the catheter and the entry site of this hard-to-approach calcified leak. In order to overcome the problem, we changed the strategy and advanced the MP from the right radial artery while the angiographic catheter was advanced from the right femoral artery. Severe paravalvular aortic regurgitation with a high burden of calcium between the prosthesis frame and native tissue was demonstrated by left and right anterior oblique (LAO/RAO) aortography (Figures 2A,B). A hydrophilic 0.35 inch-260 cm Terumo guide wire was successfully advanced across the leak into the left ventricle (LV); however, none of the catheters used (AL2- 4Fr, Judkins right 5-Fr, Multipurpose 5-Fr) could be advanced. Finally, a Glidecath 4-Fr could cross the leak over the hydrophilic wire. The hydrophilic guidewire was replaced with a stiffer guidewire (GWBC30, Medtronic Confida™) which, after creating a loop in the LV, was advanced across the self-expandable valve into the descending aorta where it was snared and externalized through the left femoral artery, thus creating an arterio-arterial loop (Figure 2C).

**Figure 3:**
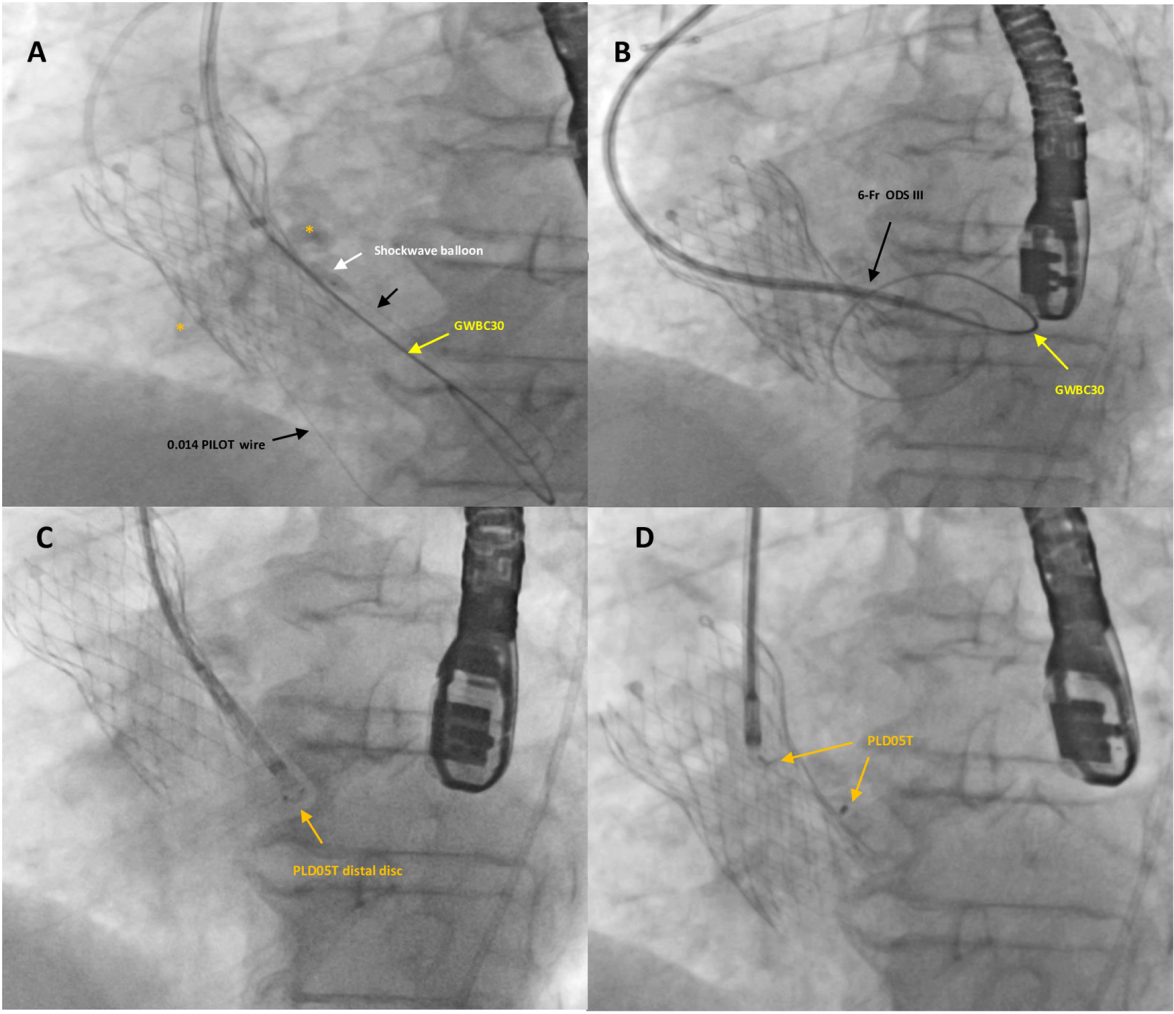
Fluoro-angiographic intra-procedural steps (2). A Shockwave balloon C^2^ IVL 3 × 12 mm (white arrow) was positioned within the leak and inflated with low pressure to prevent annular rupture (**A**). The application of the IVL to the calcified leak and the increased support provided by the Confida guidewire finally allowed the progression of the 6-Fr dedicated delivery sheath (Occlutech ODS III) into the left ventricle (**B**). A specifically designed, 5 mm square twist device (PLD, Occlutech, Helsingborg, Sweden) (orange arrows) was successfully deployed and released within the leak (**C**,**D**).

A 6-Fr Multipurpose guiding catheter was advanced over the exchange wire toward the entry of the leak, but it was impossible to reach the LV. Subsequently, the leak was crossed with an additional 0.0014 coronary guidewire (PILOT, Abbott Vascular) advanced into the LV and then into the aorta, predilated with NC Euphora™ 2 × 15 mm (Medtronic) and Accuforce 4 × 20 mm (Terumo) non-compliant balloon dilatation catheters at 12 atm to engage the PVL with the Shockwave balloon (Figure 2D). Due to the heavy concentric calcifications within the leak and in order to avoid annular rupture, a Shockwave Lithotripsy balloon 3.0 × 12 mm was positioned within the leak and inflated at low pressure for a total of 80 pulses during two shockwave cycles (Figure 3A). No hemodynamic changes occurred during the manipulation.

The application of IVL for the highly calcified leak and the increased support provided by the Confida guidewire finally allowed the 6-Fr dedicated delivery sheath (ODS III, Occlutech) to advance into the LV (Figure 3B). A specifically designed, 5 mm ST PLD was successfully deployed within the leak (Figures 3C,D). The distal disk of the PVL occluder was pushed back in order to be located within the leak (not exposed into the LV) and then, while keeping tension on the delivery cable, the proximal disk was opened in the remaining portion of the track with a sort of “elongated” shape and a stable position. After a careful “push-and-pull” maneuver, the device was successfully released and fluoroscopic and 2D TEE color Doppler showed the device within the leak tract, without interference with surrounding structures.

**Figure 4:**
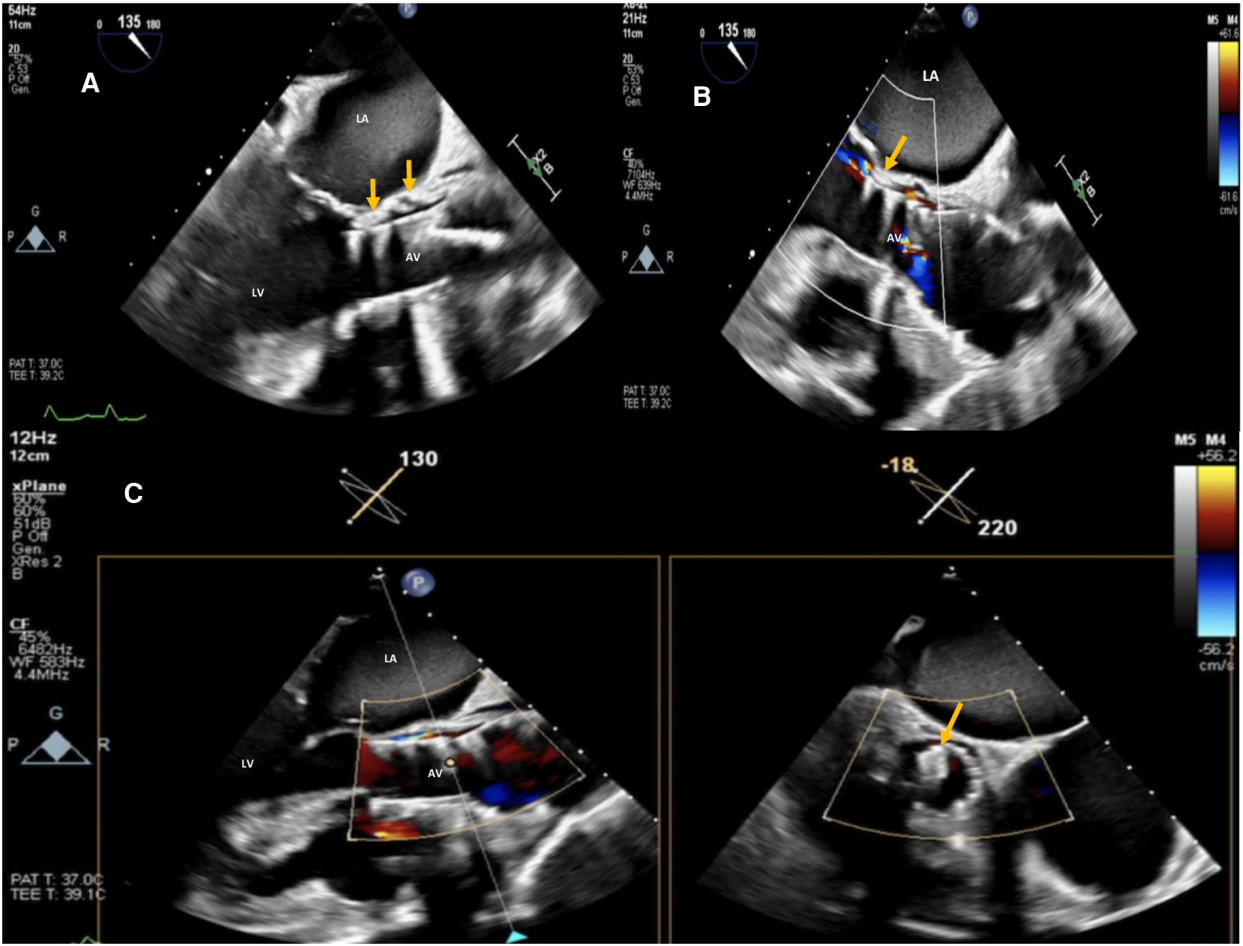
Post-procedure 2D TEE color Doppler in the mid esophageal AV long-axis view (**A,B**) and 2D X-plane TEE AV long-axis/short-axis views (**C**) showing SE TAVI (26 mm evolut™ R®) with trivial/mild residual intra-leak regurgitation and the correct position of the 5 mm PLD deployed within the leak (orange arrows). La, left atrium; LV, left ventricle; AV, aortic valve.

The final echocardiographic and angiographic control confirmed the correct position of the device within the long leak with mild residual regurgitation (Supplementary Video S4). The postoperative course was uneventful, and the patient was discharged on postoperative day 3. Follow-up 2D TEE color Doppler 1 and 3 months following the procedure confirmed the stable position of the device with trivial residual leak (Figure 4). Clinical improvement with optimal medical therapy and a better quality of life were finally achieved.

## Discussion

TAVI is a well-established technology for the treatment of patients with severe symptomatic aortic stenosis at high or prohibitive surgical risk. More recently, TAVI has become a viable alternative even in patients with intermediate-to-low surgical risk, bicuspid aortic valve disease, and aortic regurgitation (7). TAVI patients are usually older adults with a high number of comorbidities such as frailty, cognitive impairment, disability, and bleeding risk.

Significant PVL regurgitation after implantation of first-generation/second-generation TAVI devices represents a serious complication with adverse impacts on short- and long-term clinical outcomes (8). Depending on the valve and the series (9), the incidence of moderate or severe PVL regurgitation after TAVI has been reported to be up to 25%, with a much higher influence after the use of SE valves compared to BE valves (2, 10), probably due to an insufficient immediate seal. However, the incidence of PVL regurgitation has recently decreased with the development of third-generation TAVI devices with special skirts and new cuffs in order to better seal the annular region. Transcatheter closure of PVL regurgitation after TAVI may be an effective and less-invasive treatment option for these high-risk patients.

The most challenging problems are multiple or irregular leaks, low crossing positions over frame struts, and high sealing skirts on SE valves; conversely, strut avoidance is way more easier in BE valves. It is worth mentioning that device-landing-zone calcium volume (3) plays a fundamental role in the development of PVL regurgitation after TAVI, particularly after the implantation of SE devices, as demonstrated in our case.

In the majority of cases, SE valves require wire and catheter advancement through the valve struts and their manipulation can be challenging. Generally, it is difficult to advance delivery catheters, so the creation of an AA loop through the leak can be very helpful to cross through the SE struts and overcome the inner leak resistance (4, 5). In the majority of patients, the use of an additional coronary wire (buddy wire) can help advance non-compliant balloon dilatation catheters with the aim of enlarging the hard-to-approach leakage.

The so-called “Tootsie Roll Technique” of sequential deployment of a covered stent and vascular plug, which may effectively treat transcatheter heart valve-related PVL has recently been reported in the literature. In fact, a covered self-expandable stent (Viabahn, Gore®) is deployed in the defect to create a seal between the TAVI and the annulus, and then a vascular plug is deployed within the covered stent to obliterate the leakage (11). Notwithstanding, this technique would not add any benefit in our case due to the tortuosity and small size of the leak.

One more challenging finding is the severely calcified long path with high resistance or inability to advance large delivery sheaths. IVL technology has been successfully applied to coronary and peripheral calcified lesions in order to modify vessel calcifications with mechanical pressure waves, leading to significant luminal gain and facilitating optimal stent expansion. Ongoing clinical studies will add further insight into the safety and effectiveness of this technology in coronary arteries in comparison with the currently used high-pressure balloon dilatation or adjunctive atherectomy techniques (12, 13). Additionally, the delivery of shockwaves to debride the large calcific nodules present in the natural valves or even in implanted biological valves is in pre-clinical testing and should be validated in terms of long-term biological effects (14, 15).

The first description of IVL application in the treatment of calcified posterolateral PVL after a mitral surgical valve was recently published (16). Based on this, we therefore successfully applied IVL technology as an off-label use and this report constitutes the first-in-man use of lithotripsy in the treatment of a severely calcified long leakage with severe regurgitation after SE TAVI (26 mm SE Evolut™ R®).

In conclusion, balloon leak dilation followed by intravascular lithotripsy allowed us to cross the small tortuous calcified leak with the 6-FR ODS III and successfully complete the PVL closure procedure.

## Data Availability

The original contributions presented in the study are included in the article/Supplementary Material, further inquiries can be directed to the corresponding author.

## Supplementary material

Supplementary Video S1 Mid-esophageal 5 chamber view 2D TEE color Doppler showing moderate to severe PVL regurgitation after SETAVI (26 mm Evolut™ R®).

Supplementary Video S2 Mid-esophageal AV long-axis view 2D TEE color Doppler showing moderate-severe PVL regurgitation through a long, tortuous, heavily calcified leak located below the left coronary sinus after SE TAVI (26 mm Evolut™ R®).

Supplementary Video S3 Pre-procedural aortography with a Pig-tail 5-Fr catheter in left anterior oblique view showing a 26 mm SE Evolut™ R® with severe regurgitation due to a highly calcified long paravalvular leak.

Supplementary Video S4 Post-procedure aortography with a Pig-tail 5-Fr catheter in left anterior oblique view showing the correct position within the long leak of the 5 mm ST PLD with mild residual regurgitation. Note the large calcific nodules in the aortic annular region.
