# Supplementary figures and images for: Intravascular lithotripsy (IVL) enabled the percutaneous closure of a severely calcified paravalvular leak regurgitation following implantation of a self-expandable transcatheter aortic valve: a case report
Source: Front Cardiovasc Med. 2024 Feb 21;11:1359711. doi: 10.3389/fcvm.2024.1359711 (PMC10914981; doi:10.3389/fcvm.2024.1359711)

## Slide 1
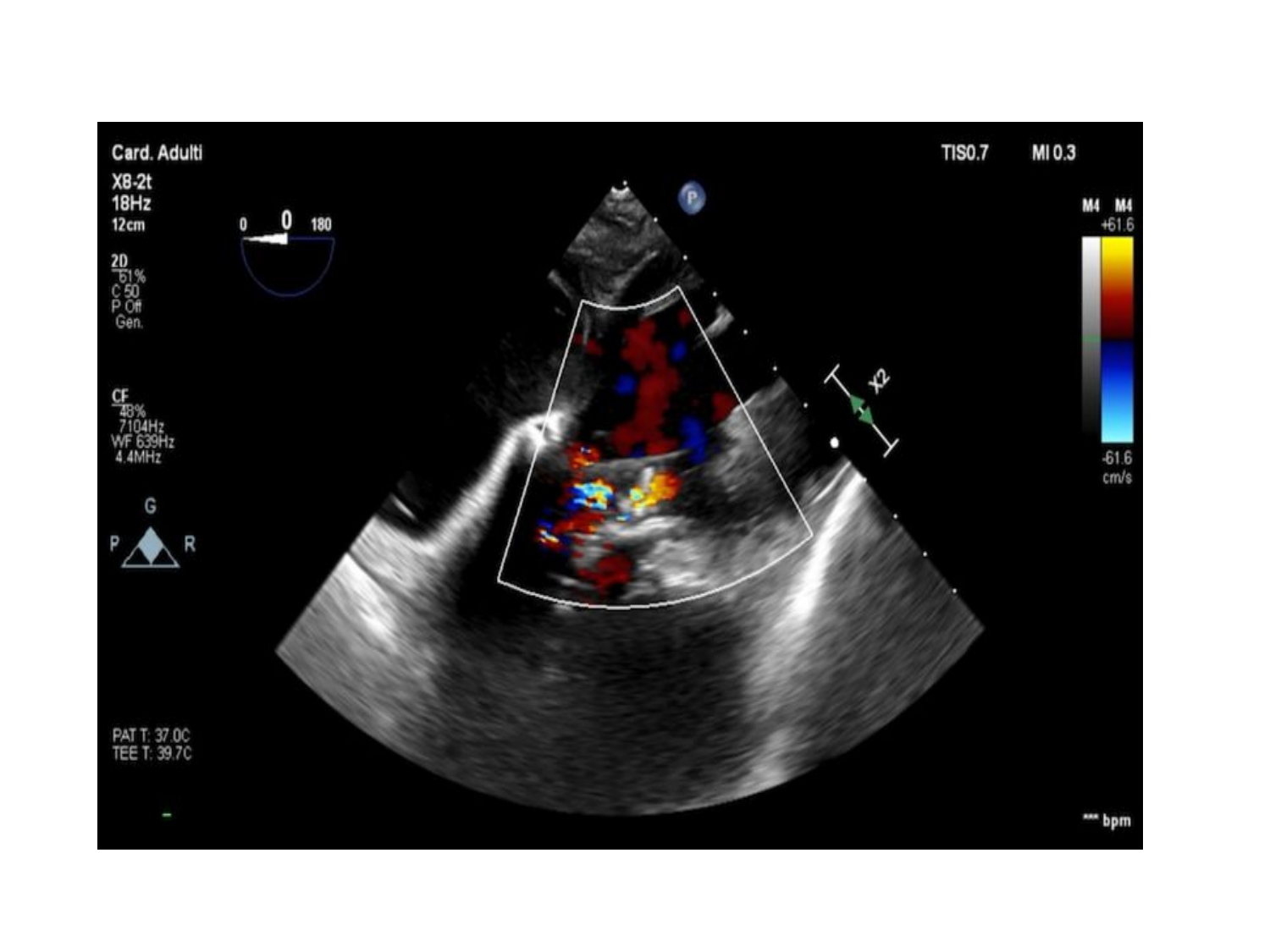

LA

Supplement: Supplementary Video S1 — Mid-esophageal 5 chamber view 2D TEE color Doppler showing moderate to severe PVL regurgitation after SETAVI (26 mm Evolut™ R®). [file Datasheet1.zip › Data Sheet 1_v1/Suppl Video 1.pptx]

## Slide 1
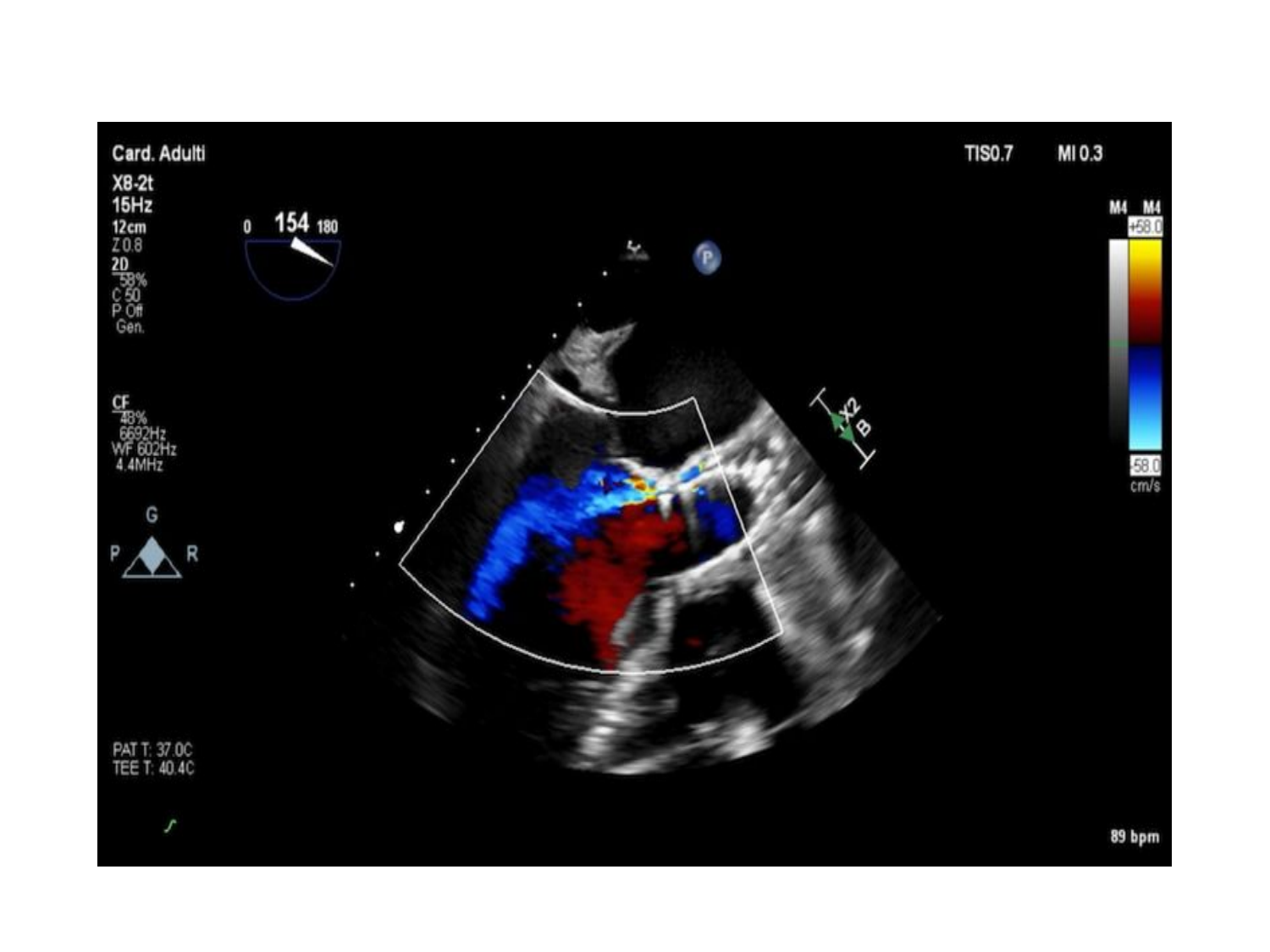

LA

Supplement: Supplementary Video S2 — Mid-esophageal AV long-axis view 2D TEE color Doppler showing moderate-severe PVL regurgitation through a long, tortuous, heavily calcified leak located below the left coronary sinus after SE TAVI (26 mm Evolut™ R®). [file Datasheet2.zip › Data Sheet 2_v1/Suppl Video 2 .pptx]

## Slide 1
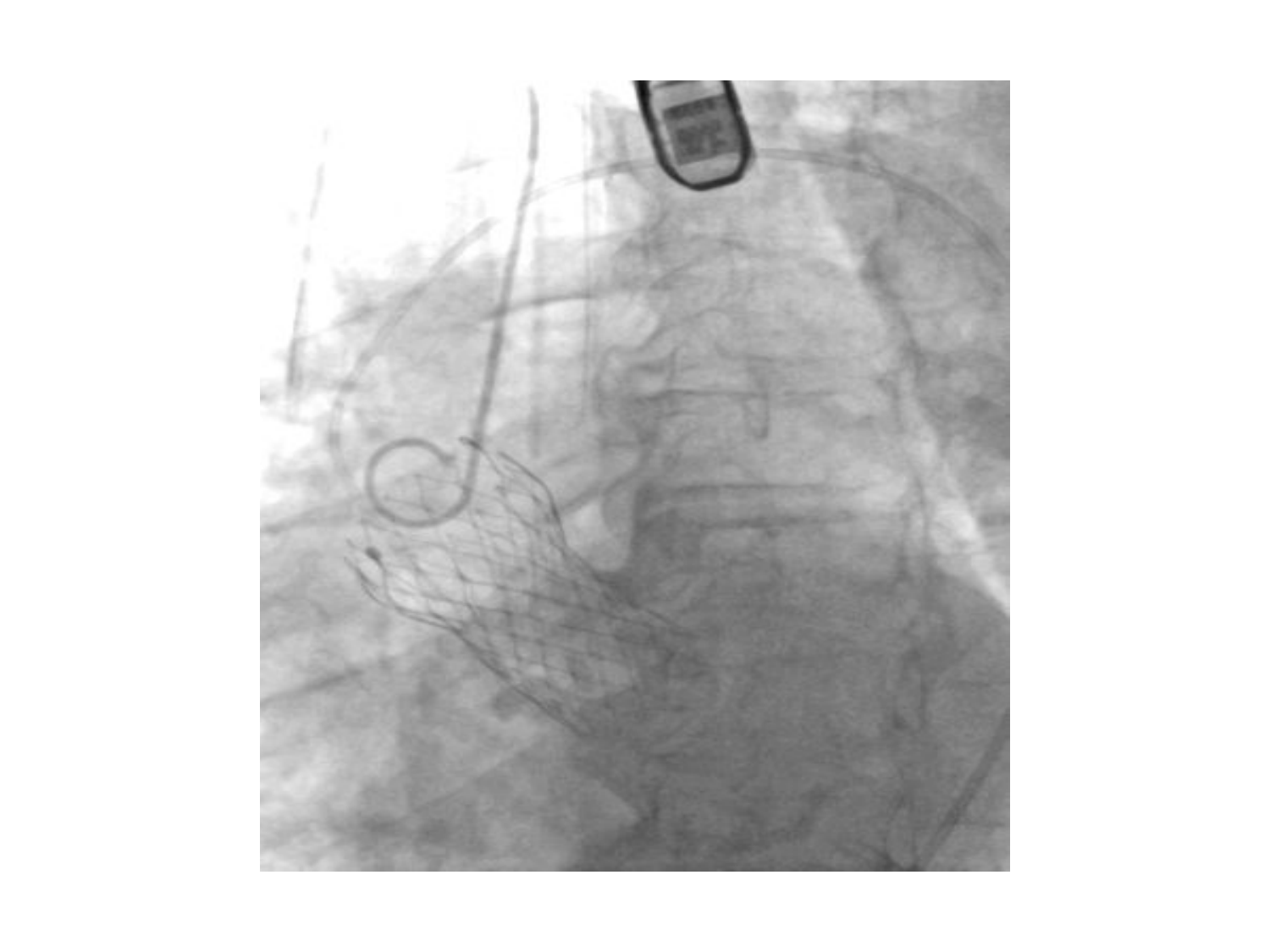

LA

Supplement: Supplementary Video S3 — Pre-procedural aortography with a Pig-tail 5-Fr catheter in left anterior oblique view showing a 26 mm SE Evolut™ R® with severe regurgitation due to a highly calcified long paravalvular leak. [file Datasheet3.zip › Data Sheet 3_v1/Suppl Video 3 .pptx]

## Slide 1
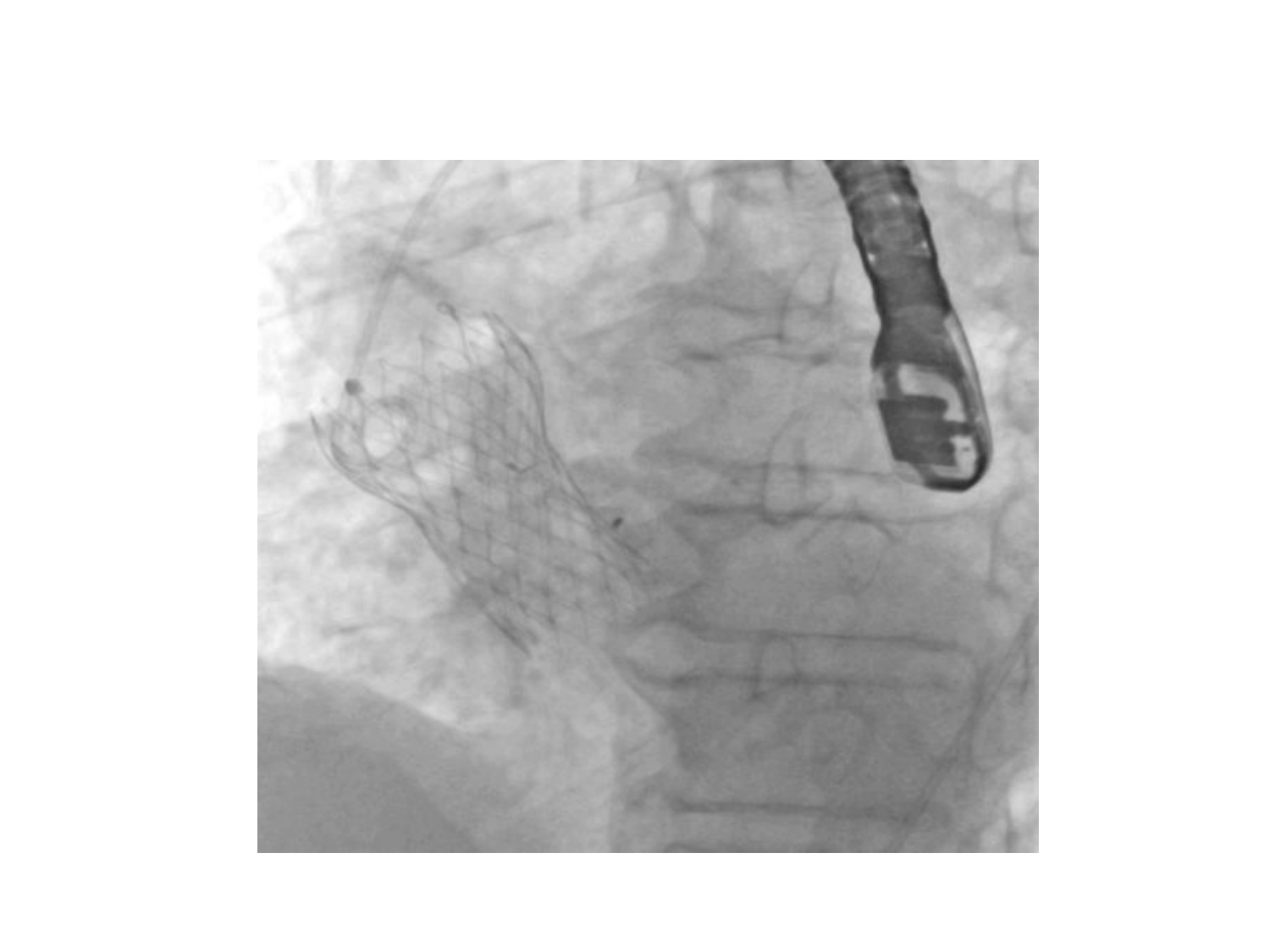

LA

Supplement: Supplementary Video S4 — Post-procedure aortography with a Pig-tail 5-Fr catheter in left anterior oblique view showing the correct position within the long leak of the 5 mm ST PLD with mild residual regurgitation. Note the large calcific nodules in the aortic annular region. [file Datasheet4.zip › Data Sheet 4_v1/Suppl Video 4.pptx]
