# Supplementary material for: Intravascular lithotripsy (IVL) enabled the percutaneous closure of a severely calcified paravalvular leak regurgitation following implantation of a self-expandable transcatheter aortic valve: a case report
Source: Front Cardiovasc Med. 2024 Feb 21;11:1359711. doi: 10.3389/fcvm.2024.1359711 (PMC10914981; doi:10.3389/fcvm.2024.1359711)
Supplement: Supplementary file 5 [file Datasheet5.pdf]

TIMELINE

October 2021

- Dyslipidemia, hypertension, atrial fibrillation on edoxaban
- Severe symptomatic aortic stenosis

January 2022

- Congestive heart failure NYHA III on optimal medical therapy
- Heart team approval of percutaneous closure for the unaccetable surgical risk

April 4, 2022

- Transcatheter self-expandable aortic valve **26 mm Evolut™ R®** (Medtronic Inc., MN, USA) implantation with mild-moderate residual leak

April 7, 2022

- Cardiac resynchronization therapy device (**CRT**, Medtronic) implantation complicated by infection, decubitus ulcer and left upper limb thrombosis. The device was then removed

December 2022

- 2D TTE/TEE color Doppler: to moderate-severe PVL regurgitation through a 10 mm long and 4 mm in diameter, tortuous, heavily calcified leak located underneath the left coronary sinus

November 2023

- Balloon leak dilation followed by intravascular lithotripsy (IVL) allowed us to get across the calcified leak with the 6-FR delivery sheath with a successfully implantation of a specifically designed, 5 mm square twist device (**PLD**, Occlutech, Helsingborg, Sweden)

December 2023

- 1-month follow-up TTE confirmed stable position of the device with trivial residual leak.
- Improvement of clinical conditions and quality of life
